# Editorial: Advancing multidisciplinary approaches in skull-base tumor management

**DOI:** 10.3389/fonc.2026.1844620

**Published:** 2026-04-14

**Authors:** Domen Vozel, Saba Battelino, Cvetka Grašič Kuhar, Jure Urbančič

**Affiliations:** 1 Department of Otorhinolaryngology and Cervicofacial Surgery, University Medical Centre Ljubljana, Ljubljana, Slovenia; 2 Faculty of Medicine, University of Ljubljana, Ljubljana, Slovenia; 3 Department of Medical Oncology, Institute of Oncology Ljubljana, Ljubljana, Slovenia

**Keywords:** endoscopic surgery, lateral skull base surgery, meningioma, nasopharyngeal cancer (NPC), pituitary tumors, sinonasal cancer, skull base, vestibular schwannoma

The management of skull base tumors is at the forefront of modern otolaryngology, neurosurgery, neuroradiology, radiation oncology and medical oncology, where technological innovation, anatomical complexity, and patient-centered care converge. Over the past decade, rapid advancements in endoscopic techniques, imaging and multidisciplinary management have transformed treatment for these tumors. This Research Topic contains eleven articles, which reflect this evolution, providing a thorough overview of the latest innovations and emphasizing the ongoing shift towards precision treatment and care driven by functional outcomes.

State-of-the art management of skull base tumors is the result of collaboration among multiple specialties, particularly otolaryngology, neurosurgery, neuroradiology, radiation oncology and medical oncology. The expertise of a single specialist from each of these fields is therefore no longer sufficient to ensure optimal treatment outcomes in the modern management of skull base tumors. This is because the skull base is an anatomical region where each specialist interprets and manages skull base tumors differently (**Table 1**).

**Table 1:** Stereotypical thought patterns in the management of skull base tumors by specialty.

Specialty	Approach
Otolaryngologist	How to ensure adequate exposure of the tumor for resection and reconstruct the skull base
• Rhinologist	How to preserve facial appearance, sense of smell, breathing, vision, and drainage of the paranasal sinuses using endoscopic approaches?
• Otologist	How to preserve facial appearance and function, hearing, balance, and taste using microscopic approaches through the middle ear?
Neurosurgeon	How to completely remove an intracranial tumor while preserving vital blood vessels and nerves
Radiation oncologist and medical oncologist	How to improve patient survival with a combination of non-surgical and surgical treatments?
Neuroradiologist	Where exactly is the tumor located in relation to anatomical structures?

The importance of a multidisciplinary approach, including phoniatric care, is also well illustrated by the study by Wang et al., which, due to the high risk of swallowing disorders, recommends screening for swallowing dysfunction and early postoperative swallowing assessment in patients scheduled for treatment of posterior skull base tumors.

Pathologists play a very important role in the management of tumors, as the sinonasal region is the most histologically diverse anatomical area in the human body. In the past, the diagnosis of sinonasal undifferentiated carcinoma (SNUC) was common; today, however, due to more precise molecular-genetic diagnostics, this diagnosis is made less frequently, with the identification of new types of cancer, such as SWI/SNF complex-deficient sinonasal carcinoma. Diagnosis can be challenging, particularly when only slightly more than 100 cases have been described in the literature, as is the case with the adult atypical teratoid rhabdoid tumor described in the article by Hernandez-Rovira et al.

The result of multidisciplinary collaboration in the field of skull base surgery is strongly reflected in the transition of surgical approaches from predominantly classical (i.e., microscopic open) approaches to the skull base to endoscopic approaches, which have been adopted over the past three decades from transnasal endoscopic skull base surgery (Han et al.; Xie et al.; Felbabić et al.; Shen et al.). The endoscopic view, among other things, allows for sufficient magnification, an angled view, and flexibility in directing the view through the body’s natural orifices (e.g., the nose) or through smaller craniotomies based on the principle of keyhole surgery. The endoscopic approach is the method of choice for tumors that were once treated exclusively with microsurgical techniques, such as clival chordomas and posterior skull base tumors. Recovery is also faster following endoscopic approaches compared to open surgery. Endoscopic techniques can also be usefully applied in the treatment of deep brain lesions, such as intraventricular hemorrhage (Shafiq et al.)

Regardless of the successes of surgical treatment as part of multimodal therapy, it is important to recognize the effectiveness of non-surgical treatment options, including the development of non-surgical methods such as radiation therapy and systemic therapy. The good efficacy of non-surgical therapy is well demonstrated in the article by Albilali et al., which describes the recovery of function in several cranial nerves following damage caused by extensive nasopharyngeal cancer. Undoubtedly, this is not possible with surgical treatment; therefore, surgical treatment of nasopharyngeal cancer is still indicated as primary therapy only for selected histological types (e.g., mucosal melanoma, minor salivary gland carcinomas) or as curative treatment for cancers in which the tumor could be microscopically resected into healthy tissue with acceptable morbidity.

Advances in radiological techniques, knowledge of surgical anatomy and surgical techniques, as well as non-surgical treatment methods, are enabling us to treat increasingly extensive tumors that were once considered incurable or unresectable. A classic example of the resectability of tumors once considered unresectable are tumors of the infratemporal and pterygopalatine fossae. With open approaches to these anatomical regions, control of surgical margins was poor and resectability was questionable. A good demonstration of the resectability (88%) of these tumors is presented in the study by Zoli et al. By applying other non-medical expertise, it is possible to predict the resectability of certain tumors based on preoperative imaging diagnostics, as demonstrated by Shen et al. using a mathematical model to determine the resectability of pituitary adenomas. The field of skull base tumors is becoming well-supported by scientific evidence, despite the fact that these are rare diseases. This is evidenced by the studies by Zoli et al., Shen et al., and Li et al. in this Research Topic, which include large patient series.

For skull base tumors, there are often no internationally standardized decision-making algorithms for management, and clinical guidelines are frequently inapplicable. In some cases, these guidelines may even conflict with established treatment approaches for skull base tumors; for example, the National Comprehensive Cancer Network (NCCN) guidelines for the management of locally very advanced (cT4b) sinonasal cancer (1). According to the NCCN guidelines, locally very advanced sinonasal cancer would always be considered a disease treated non-surgically (1). On the other hand, certain diseases are most effectively treated surgically, regardless of stage. This field is subject to significant treatment individualization and dynamic treatment decision-making, including based on preoperative and intraoperative findings, as reflected in the articles by Shen et al. and Felbabić et al.

One of the cornerstones in the management of skull base tumors is a multidisciplinary team, which serves as the authoritative body within a healthcare institution for the care of such patients (2). In most institutions, the treatment of skull base tumors represents a smaller but significant part of the overall clinical practice of each member of the multidisciplinary team. For example, an otolaryngologist, who primarily treats sinonasal tumors and inflammatory conditions, manages skull base tumors several times a week. The same applies to other specialists on the multidisciplinary team. Managing a broad spectrum of diseases within one’s specialty also fosters flexible thinking in the management of skull base diseases. In our opinion, any patient with a skull base tumor who is not treated in a multidisciplinary manner is deprived of the best possible care. Multidisciplinarity must be strengthened through educational programs in the field of skull base tumors and collegial relationships among specialists from different disciplines.
